# Whole spectrum of *Aeromonas hydrophila* virulence determinants and the identification of novel SNPs using comparative pathogenomics

**DOI:** 10.1038/s41598-023-34887-1

**Published:** 2023-05-12

**Authors:** Bahaa Abdella, Nourhan A. Abozahra, Nermeen M. Shokrak, Radi A. Mohamed, Ehab R. El-Helow

**Affiliations:** 1 Aquaculture Department, Faculty of Aquatic and Fisheries Sciences, Kafrelsheikh University, Kafrelsheikh, 33516 Egypt; 2 Department of Botany and Microbiology, Faculty of Science, Alexandria University, Alexandria, 21511 Egypt

**Keywords:** Bacteria, Bacteriology

## Abstract

*Aeromonas hydrophila* is a ubiquitous fish pathogen and an opportunistic human pathogen. It is mostly found in aquatic habitats, but it has also been isolated from food and bottled mineral waters. It causes hemorrhagic septicemia, ulcerative disease, and motile *Aeromonas* septicemia (MAS) in fish and other aquatic animals. Moreover, it might cause gastroenteritis, wound infections, and septicemia in humans. Different variables influence *A. hydrophila* virulence, including the virulence genes expressed, host susceptibility, and environmental stresses. The identification of virulence factors for a bacterial pathogen will help in the development of preventive and control measures. 95 *Aeromonas* spp. genomes were examined in the current study, and 53 strains were determined to be valid *A. hydrophila*. These genomes were examined for pan- and core-genomes using a comparative genomics technique. *A. hydrophila* has an open pan-genome with 18,306 total genes and 1620 genes in its core-genome. In the pan-genome, 312 virulence genes have been detected. The effector delivery system category had the largest number of virulence genes (87), followed by immunological modulation and motility genes (69 and 46, respectively). This provides new insight into the pathogenicity of *A. hydrophila*. In the pan-genome, a few distinctive single-nucleotide polymorphisms (SNPs) have been identified in four genes, namely: d-glycero-beta-d-manno-heptose-1,7-bisphosphate 7-phosphatase, chemoreceptor glutamine deamidase, Spermidine N (1)-acetyltransferase, and maleylpyruvate isomerase, which are present in all *A. hydrophila* genomes, which make them molecular marker candidates for precise identification of *A. hydrophila*. Therefore, for precise diagnostic and discrimination results, we suggest these genes be considered when designing primers and probes for sequencing, multiplex-PCR, or real-time PCR.

## Introduction

Aquaculture is one of the fastest-growing food industries that supplies the world with high-quality proteins. In 2020, 122.6 million metric tonnes of aquaculture products were produced globally, with a market value of USD 281.5 billion^1^. The increase in aquaculture production helps in minimizing the gap formed due to the food shortage, which is caused by overpopulation. Therefore, the quantity of farmed fish from both marine and freshwater farms has expanded considerably during the last five decades^2^. In the last 20 years, world aquaculture's yearly production increased by 609% between 1990 and 2020, with an average annual growth rate of 6.7%^1^. One of the successful freshwater aquaculture candidates is the Nile tilapia (*Oreochromis niloticus*), which is cultivated in several regions of the world, including China, Indonesia, and Egypt as the top three producing countries^3^. The increasing output necessitates the efficient use of water resources, which has been achieved through intensive aquaculture systems; however, it is a very stressful environment for cultivated animals and has intensified the development and spread of bacterial infections^4^. Freshwater bodies have been found as reservoirs of *Aeromonas hydrophila*, which may infect fish, reptiles, amphibians, bivalves, and humans^5–8^. It has been proven to be the causative agent of numerous disease outbreaks in fish, including septicemia, ulcerative and hemorrhagic diseases^2,9^.

*Aeromonas hydrophila* is also a public health concern because of its ability to transfer illness to humans since it has been isolated from a variety of sources, including food, animals, groundwater, and wastewater, in different stages of treatment^10–12^. Therefore, it is crucial to precisely diagnose and identify bacterial strains belonging to that species. However, phenotypic methodologies were applied for the characterization of *Aeromonas* spp. resulted in erroneous and inconsistent results^13^. For instance, after preliminary phenotypic identification of 119 strains isolated from sick fish, only 35.5% were verified to the species level as *A. hydrophila* using 16S rRNA-RFLP and *rpoD* sequences^14^. Although 16S rRNA gene sequencing is frequently applied for bacterial classification, it has limited discriminatory power at the species level^13^. On the other hand, several distinct virulence genes have been identified so far as sharp molecular chronometers applicable for diagnostic purposes^15^. However, to the best of our knowledge, there are a few earlier studies concerned with virulence gene-based typing of *A. hydrophila*.

Practically, *A. hydrophila* identification is mostly based on the presence of virulence genes such as cytotoxic enterotoxin gene (*act*), aerolysin gene (*aer*), hemolysin A gene (*hylA*), and enterotoxin gene (*ast*)^16^. However, some of these genes are present in non-*A. hydrophila* strains, and it is not necessary to detect all virulence genes in a single pathogenic strain; as evidenced by several investigations employing either fish or clinical samples^17–19^. Therefore, and because of the limitations inherent in such methods, both the 16S rRNA sequence and the PCR detection virulence genes may misidentify novel isolates.

For infection control in fish farms, the perfect vaccine is not yet available due to the genetic diversity of *Aeromonas* strains as well as the lack of a universal biomarker gene that could be used for molecular typing or as a vaccine target. Many approaches have been applied for infection control, including the use of antibiotics which has the drawback of increasing antimicrobial resistance^17,20,21^. In other words, due to a shortage of understanding of the genetic diversity of *Aeromonas*, vaccines can only provide limited protection^17^. The lack of cross-protection against various isolates may be the reason why a commercial vaccine against *A. hydrophila* infection hasn’t yet been developed. Serotypes are also imperfectly defined, which can cause mutants to escape and reinfect animals who have received vaccinations. Moreover, effective diagnostic tests that accurately distinguish between “target” and “non-target” pathogens might facilitate their infection control^22^.

It is also important to highlight that the virulence factors mentioned in the literature were mostly based on investigations conducted on *A. hydrophila* SSU, which was later reclassified as *Aeromonas dhakensis*^19^. For molecular typing of bacterial species, multi-locus sequence typing (MLST) has proven its reliability in strain identification, by using housekeeping genes such as *rpoD* in combination with 16S rDNA-RFLP^14^. However, those methods still have considerable limitations, such as the cost and computing resources needed.

Recently, complete genome sequencing technologies have greatly improved phylogenetic analysis, genotyping and bacterial identification, antibiotic genes assessment, and disease surveillance. In an attempt to validate the publicly available genomes of *A. hydrophila* and to identify the total virulence factors they carry, 14 full *A. hydrophila* genomes and 51 draft genomes were investigated in 2018^23^. However, the number of full genomes has now grown more than thrice, and it is critical to shed more light on the virulence potential of *A. hydrophila* to remove the current ambiguities in its classification and purpose new effective molecular markers for correct identification.

It is known that virulence factor acquisition and antibiotic resistance genes are mainly happening through horizontal gene transfers rather than vertical transmission^22,24,25^. Such markers are useless on certain occasions and cannot reflect exact taxonomic relationships. Therefore, it is critical to update our knowledge of the virulence factor profile in *A. hydrophila* based on precisely named strains by considering the maximum number of available genotypes. In the present study, 95 complete genomes of the genus *Aeromonas* were analyzed using a comparative genomics approach. Thereafter, the pan-genome and core-genome were built, and the average nucleotide identity (ANI) was calculated. These findings supplied substantially comprehensive genetic diversity of *A. hydrophila*, offering critical information on the species genomic makeup and virulence factors. The pathogenicity potential of *A. hydrophila* strains was investigated, and new SNPs markers for *A. hydrophila* differentiation were explored.

## Materials and methods

### Genomic dataset of *Aeromonas* spp.

A total of 95 full genome sequences of the *Aeromonas* genus were retrieved from the National Center for Biotechnology Information (NCBI). Genome completeness, strains recognized as *Aeromonas hydrophila* and *Aeromonas* sp., and accessible complete genomes from other *Aeromonas* species were employed as inclusion criteria. All complete genomes identified as *A. hydrophila* (n = 54), *Aeromonas* spp. (n = 27), and representatives of other *Aeromonas* species (n = 14) were obtained from GenBank during October 2022. Each genome's information and BioSample records were also collected. The supplementary Table S1 lists detailed information on the 95 *Aeromonas* spp. strains used in this study, including the accession number of each data set and the download link. Only complete genomes were considered to provide the most realistic picture of the strain’s virulence gene diversity and potential identification biomarkers.

### Average nucleotide identity and digital DNA-DNA hybridization

For the confirmation of species affiliations, nucleotide-level comparisons of each pairwise combination of genomes were conducted using average nucleotide identity (ANI). The Python script PyANI v0.2.7^22^ was used to compute pairwise ANI values using two different methods, the MUMmer^26^ and the BLAST+ method^27^ employing the ANIm and ANIb options, respectively. The valid members of *A. hydrophila* have been selected for further analysis, and the misidentified strains were excluded. This was based on the threshold value that indicates the presence of the same species of ANI being greater than 94%^22,28^. For confirmation of ANI results, the recommended BLAST+ method was utilized to create digital DNA-DNA hybridization (dDDH) values for DNA-DNA hybridization using the Genome-to-Genome Distance Calculator online server (GGDC v3)^29^. To complete this stage, all genome sequences were presented as queries against all other sequences that had been analyzed. Formula 2 had been used to calculate the DNA-DNA hybridization scores. Formula 2 was used because of its tolerance to genome size. The cut-off value for dDDH was 70% for species discrimination.

### Core- and Pan-genome construction

To preserve consistency and uniformity, all genomes of the examined *Aeromonas* spp. strains were functionally re-annotated using the Prokka suite, version 1.14.6. The following settings were used to predict the protein-coding genes: -genus *Aeromonas* -species hydrophila -evalue 1e−09 -coverage 80 -mincontiglen 200) according to Podrzaj et al.^28^. Roary^30^ was then used to build the core- and pan-genome of *Aeromonas* spp. using a default identity threshold of 95%. Briefly, the genes have been categorized into four groups: core genes, which are present in more than 99% of genomes. Soft core genes, which are present in more than 95% but less than 99% of the strain. Shell genes, which are present in 15% to less than 95% of the strains, and cloud genes, which are present in less than 15 percent of the total number of strains. PanGP^31^ tool was used to visualize both the core and pangenomes, by using a gene presence/absence matrix generated from Roary as an input. In brief, pan-genome curve-fitting was achieved using Heaps' law, as described by Podrzaj et al.^28^. The curve equation was (y_pan_ = A_pan_ x^Bpan^ + C_pan_), where y is the size of the pan-genome, x is the number of genomes, while A, B, and C are the curve-fitting parameters. When the Bpan value falls between 0 and 1, with the progressive addition of more genomes, the size of the pan-genome grows indefinitely, suggesting an open pan-genome.

### Genes encoding virulence factors

A custom bash script was used to determine the virulence-encoding genes in *A. hydrophila* strains. Based on the virulence factor genes obtained from the virulence factor database (VFDB), the virulence factor was filtered and determined^32^. All genes that were observed only in *A. hydrophila* were identified using a dataset generated by the Roary tool’s integrated function query_pan_genome with the difference option. Following that, the virulence genes present in all valid *A. hydrophila* strains were identified. Then the differential virulence genes were found based on the presence only in valid *A. hydrophila* strains or it has similarity lower than 95% as a cut-off value of the Roary tool.

### Phylogenetic analysis and SNPs identification

Phylogenetic trees were reconstructed using 16S rRNA and uniquely determined genes. After obtaining multiple sequence alignments using MUSCLE (defaults values: Gap Open Penalties = − 400.00 /Gap Extend Penalties = 0.00) and the proper evolutionary model was chosen, MEGA-X^33^ was used for phylogenetic tree reconstruction. The trees were reconstructed using the maximum likelihood method and the most proper substitution model. All positions with coverage of less than 95% were eliminated. The bootstrap value was assigned to 500. The multiple sequence alignment CLUSTALW format was constructed using T-Coffee tools to find relevant SNPs that could be used for genotyping or molecular identification, and a customized Python script msa2snp.py (https://github.com/pinbo/msa2snp) was then used to find all SNPs in the genes of interest. The output of SNPs detection was subjected to manual curation to select the most informative one which is present only in 100% of *A. hydrophila* genomes.

## Results

### General genomic features of *Aeromonas* spp.

The overall genomic characteristics of the *Aeromonas* strains used in this investigation are shown in Supplementary Table S1. *Aeromonas simiae* A6 and *Aeromonas* sp. ASNIH4 had the smallest and largest genome sizes, respectively, and the whole genome sizes varied between 3.97 and 5.48 Mbp. The genomes that have been confirmed to be *A. hydrophila* showed genome sizes within the range of 4.30–5.45 Mbp, with *A. hydrophila* WCX23 and *A. hydrophila* KAM330 having the smallest and largest genome sizes, respectively. The number of coding sequences (CDSs) in these genomes also differed among the complete genomes of all *Aeromonas* strains (range: 3782–5161 with an overall 36.46% difference) and within the *A. hydrophila* genomes (range: 4091–5134 with an overall 25.49% difference). The total guanine and cytosine content (G + C content) of the genomes of *Aeromonas* spp. ranged between 57.9% and 62.9%, while within *A. hydrophila*, the range was 60.4–62.0%, with relatively small variations across strains. Considerable variation was also seen in the tRNA numbers among all genomes (Supplementary Table S1).

### ANI and dDDH analysis of *Aeromonas* spp.

**Figure 1:**
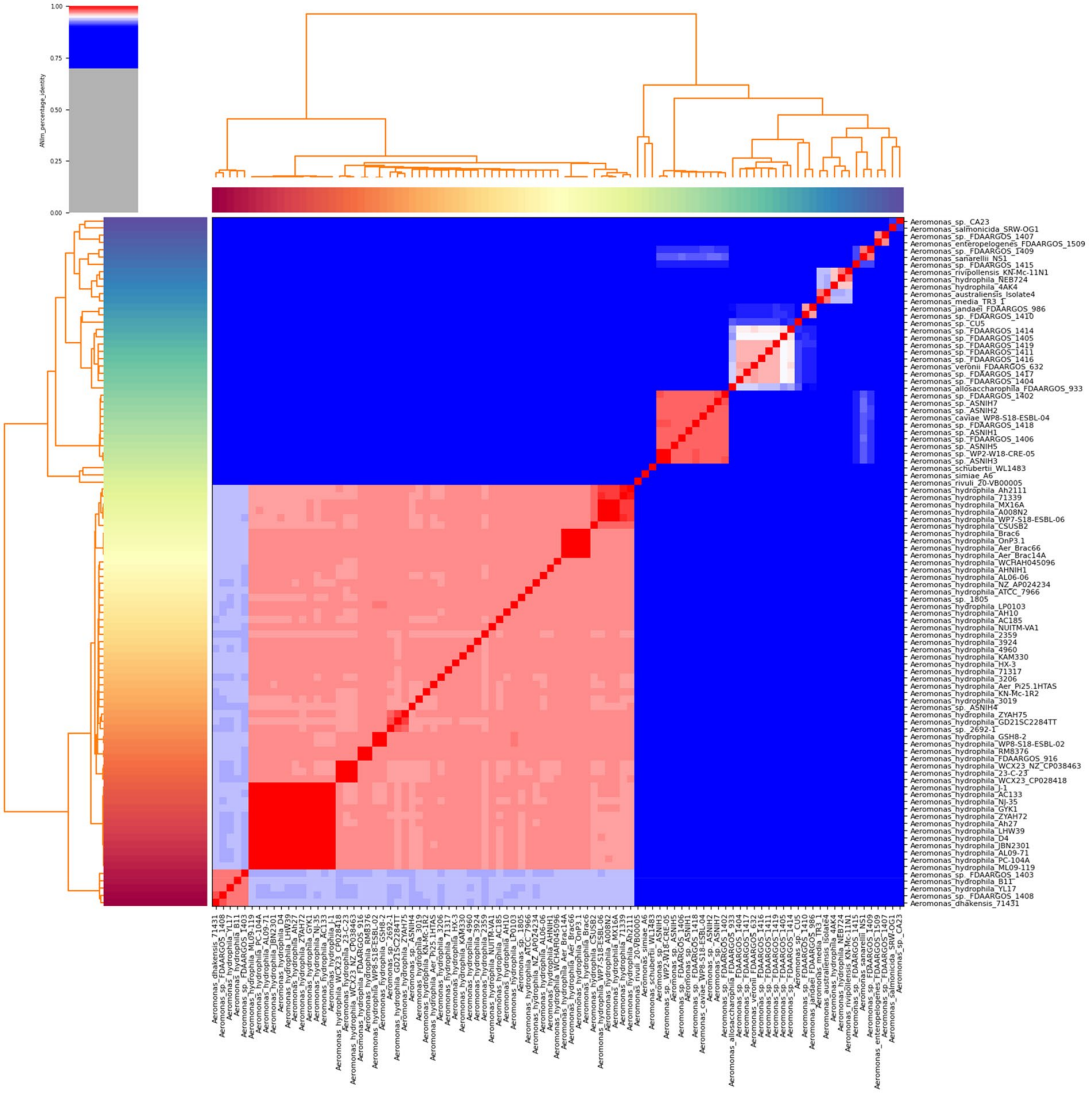
Heat map of pairwise ANI values across 95 *Aeromonas* spp. genomes. The color coding for the genomes on the x-axis and y-axis was used to differentiate the strains. Red color shows high similarity suggesting the same species while blue color shows low similarity and distinct species.

According to the result of the ANI analysis presented in Fig. 1, all strains were divided into two big clades, one of which holds most species that are known to be *A. hydrophila*. Only 53 genotypes were found and classified as *A. hydrophila* strains. This resulted from the exclusion of four strains previously classified as *A. hydrophila* and the inclusion of three strains without taxonomic species status. These include the strains NEB724, 4AK4, B11, and YL17, which are eliminated, while *Aeromonas* sp. 2692-1, *Aeromonas* sp. 1805, and *Aeromonas* sp. ASNIH4 were included and identified as *A. hydrophila* genotypes. The inclusion and exclusion were observed and consistent with both the ANI, MUMmer and BLAST+ methods. The ANIm value for excluded strains was less than 0.95 compared to the strains valid as *A. hydrophila*. While the newly included strains showed ANIm values greater than 0.95. This similarity was also confirmed by the dDDH as the excluded strains showed dDDH values of less than 70%. The included strains have values greater than 70%.

Only 12 strains showed ANIm greater than 0.99 to each other (ML09-119, PC-104A, AL09-71, JBN2301, D4, LHW39, Ah27, ZYAH72, GYK1, NJ-35, AC133, and J-1) (supplementary Table S2). ANI reveals divergence and genetic heterogeneity among *A. hydrophila* members. Consequently, it will be considerably more challenging to identify a target sequence that may be employed for diagnostic purposes. All the strains that were clustered together as *A. hydrophila* scored higher than 70%, according to the dDDH results (supplementary Table S3).

### Core- and Pan-genome analysis

**Figure 2:**
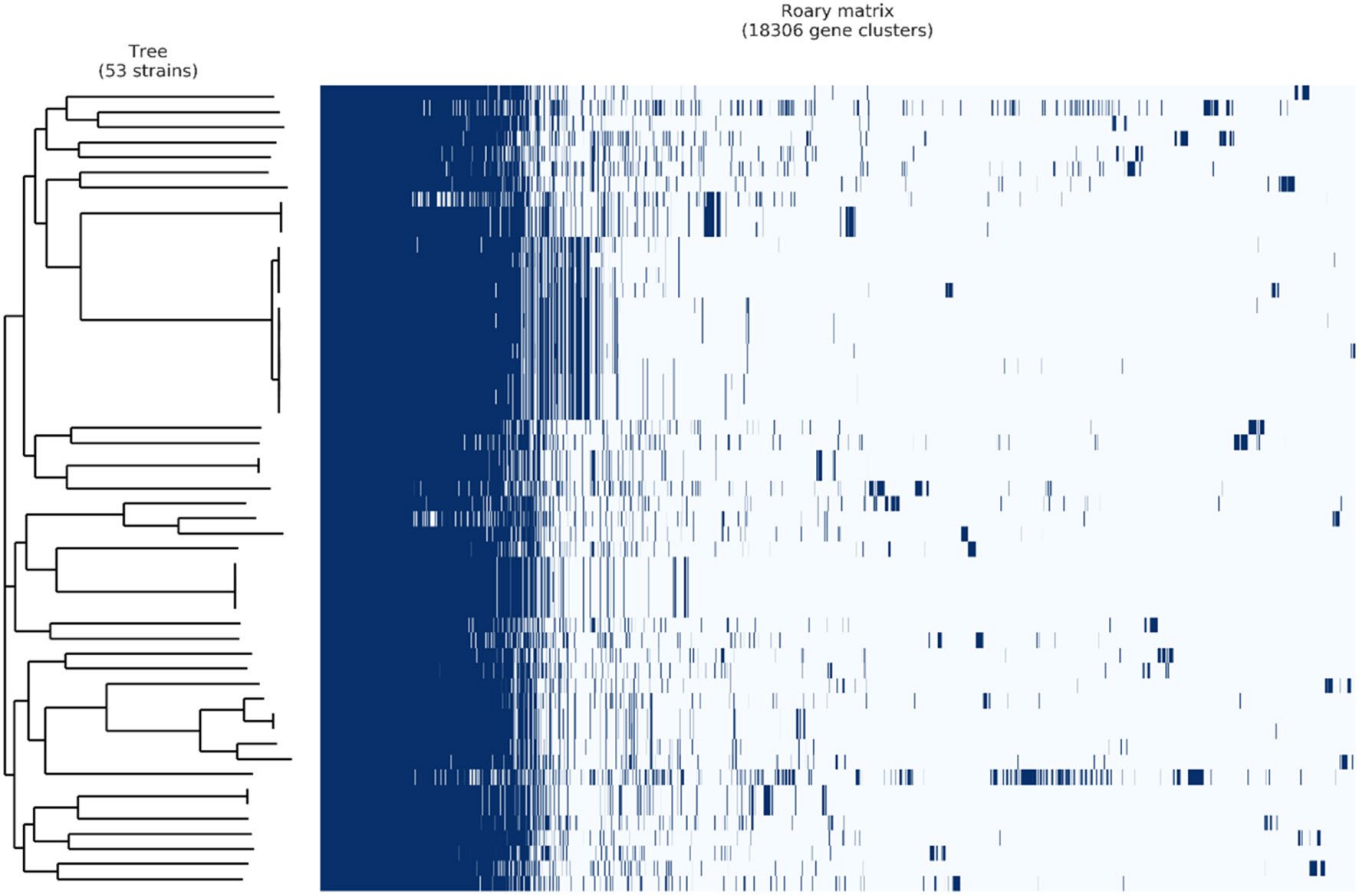
*A. hydrophila* pan-genome visualization by Roary of 53 valid genomes. The complete genomes of the strains were clustered based on the presence and absence of genes. Blue, presence of genes; white, absence of genes.

**Figure 3:**
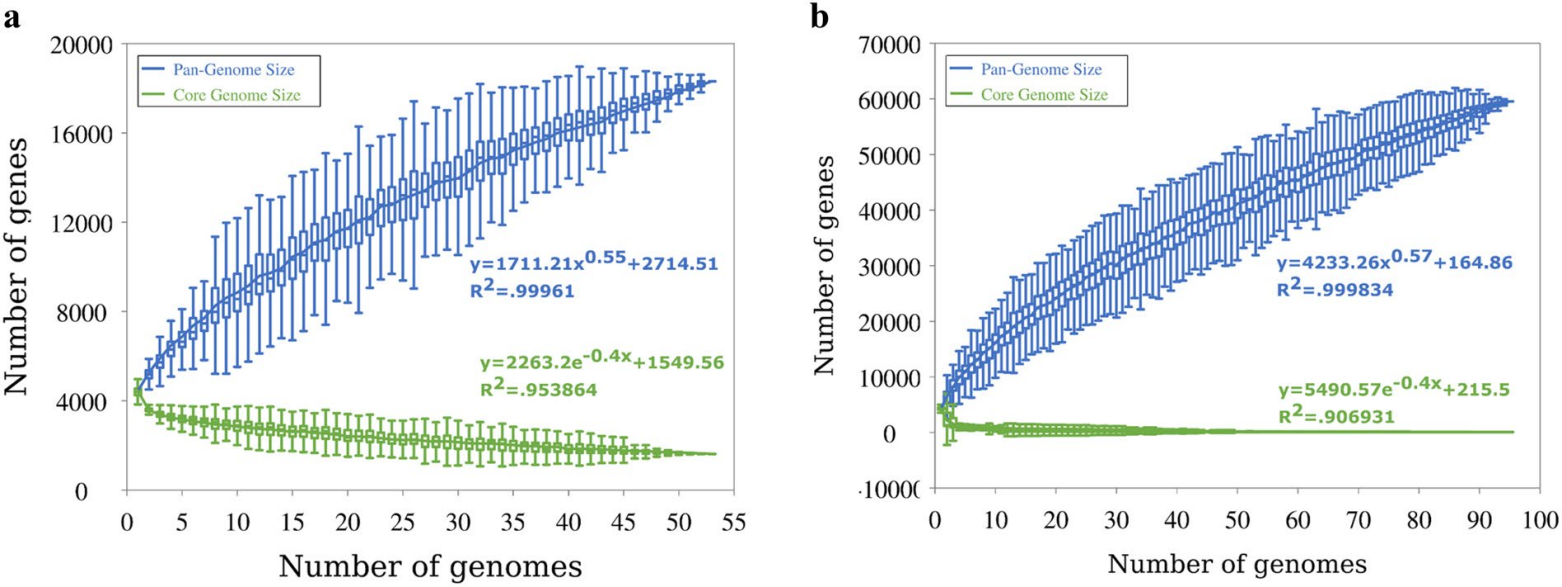
The increase and decrease of gene number in pan (blue) and core (green) genomes, respectively. (**a**) = gnomes identified as *A. hydrophila* (n = 53), (**b**): genomes of all *Aeromonas* spp. strains under investigation (n = 95). The gene accumulation curves for the power-law regression model are shown as a function of the number of consecutively added genomes.

A pan-genome study was undertaken to better understand the differences in virulence genes across *A. hydrophila* strains (Fig. 2). The figure was constructed based on the presence and absence of genes in all valid strains (n = 53). The estimated number of genes in the pan-genome was 18,306 genes. The increase and decrease of gene numbers in pan and core-genomes, respectively, are presented in Fig. 3. The analysis revealed that both the pan-genome and core-genome are open. The “B” values for the curve-fitting equations for *A. hydrophila* and *Aeromonas* spp. are 0.55 and 0.57, respectively. *A. hydrophila* has 1620 genes in its core-genome and 18,306 total genes, according to the pan-genome analysis. The number of genes in the pan-genome and core-genome was drastically altered by the addition of strains belonging to *Aeromonas* spp. The *Aeromonas* spp. When all strains were considered, the pan-genome and core genome had 59559 and 79 genes, respectively. According to the estimates, the pan-genome of *A. hydrophila* is 4.0 times greater than the average genome size of each strain (average genes number = 4577; Fig. 3a); this represents more than two folds when the entire number of strains is taken into consideration (Fig. 3b). Only 1620 genes, or 8.84% of genes, were shared by *A. hydrophila*, while only 79 genes, or 0.13%, were shared by all *Aeromonas* spp. Figure 3.

### Genes encoding virulence factors

For the determination of virulence genes in *A. hydrophila*, the Roary output was screened based on the comparison with VFDB-indexed genes. The detected genes are listed in Table 1 along with their functional categories. It is worth mentioning that most of the virulence genes are not common in all strains. For instance, the aerolysin (*aerA*) gene is detected in only 40 out of the 53 valid strains tested. Likewise, hemolysin (*hylA*) genes were found in only 48 strains. If such genes are used to name unknown strains, there would be high false negative rates of 24.5% and 9.4% for *aerA* and *hylA*, respectively. Therefore, for fast PCR detection, it is essential to name genes whose presence is restricted to *A. hydrophila* and are absent in other species. A total of 312 genes representing virulence factors in the pan-genome were found (Table 1). Overall, the effector delivery system has the largest number of genes (87) and is followed by the immune modulation factor which has 69 genes. Remarkably, only virulence genes, namely, *gmhB, and cheD,* besides *speG*, and *nagL*, have been commonly recognized in all *A. hydrophila* genotypes.

**Table 1:** The functional category of 312 virulence genes found in *A. hydrophila* pan-genome.

Gene functional category and (number of genes)	Virulence genes
Effector delivery system (87)	*ascD, bepC, bepF, bepG, clpB, clpB/vasG, clpV1, copB, cvpA, eae, epsC, epsD, epsE, epsG, epsH, epsL, epsM, epsN, exeD, exsA, exsC, flhA, glgA, gspF, gspK, hcp, hcp-1, hcp-2, hcp/tssD, hsiA1, hsiE1, hsiF1/tssE, hsiG1/tssF, hsiH1/tssG, hsiJ1, iglC/hcp, invA, lasA, lcrE/yopN, lcrO/yscI, lcrV, lepA, lepB, ligA, map, mrcA, pldB/tle5b, pscE, pscF, sdhA, sdhB, setA, sicA, sipA/sspA, spaP, spaQ, spaR, sycE/yerA, sycN, sycN/vcr2, tyeA, tyeA/vcr1, vgrG1, vipA/mglA, vipB/mglB, virB4, virB4-1, virB4-2, virB4/cagE, virG/yscW, xcpT, xcpW, xcpX, ylpB/yscJ, yopB, yopD, yscB, yscD, yscG, yscK, yscL, yscN, yscO, yscQ, yscU, yscX, yscY,*
Immune modulation (69)	*cpsB/cdsA, cysC, dep/capD, fabZ, fcl, galE, galU, glf, gmd, gmhA, gmhA/lpcA, gmhB, hisH, hldD, hldE, hscA, kdkA, kdsA, kdsB, kdtA/waaA, kpsD, kpsM, kpsT, legI, licC, lipA, lipB, lpxA, lpxA/glmU, lpxB, lpxC, lpxD, lpxH, lpxK, lpxL, lpxM, msbA, napA, neuA, neuC, per, pgi, pgm, ppsA, ppsC, pseB, pseC, pseI, pspA, rfaF, rfbA, rfbB, rfbC, rfbD, rfbF, rfbG, rfbM, rhlB, rpe, tesA, ugd, waaA, waaA/kdtA, wbpA, wbpD, wbpE, wbpI, wcaJ, wecA,*
Motility (46)	*cheD, cheZ, flaB, flaD, flgA, flgB, flgC, flgD, flgE, flgE_1, flgF, flgG, flgG_2, flgH, flgI, flgJ, flgK, flgL, flhA, flhB, fliA, fliC, fliD, fliE, fliF, fliG, fliH, fliI, fliM, fliN, fliP, fliQ, fliR, fliS, lafS, lafT, lafU, motB, pdxA, pdxJ, pflA, pseB, pseC, pseI, rpoN, ylxH*
Nutritional/Metabolic factor (36)	*barA, bauA, bauC, bioA, bioB, bioC, bioD, bioF, carA, carB, ccmA, ccmB, ccmC, ccmD, ccmE, chuW, dhbF, entA, entB, entC, entD, entE, fbpC, feoB, fepE, fyuA, fyuA/psn, ggt, hpt, iucD, mgtB, mgtC, panC, purM, pvdQ, pyrB*
Adherence (27)	*cbpA/pspC, csgD, eap/map, fbaA, fimD, focA, focC, gbpA, lepA, lpfA, lpfB, mshA, p1/MgPa/gapA, papC, papD, pilQ, pilY1, rpoN, rpoS, scpA/scpB, spaP/pac, tufA, yagV/ecpE, yagW/ecpD, yagX/ecpC, yagY/ecpB, yagZ/ecpA*
Regulation (11)	*bvgA, csrA, fur, phoP, phoQ, phoR, prfA, rcsB, relA, rpoS, sigE*
Exotoxin (10)	*aerA/act, cyaA, cyaB, hlyA, hlyB, hlyC, hlyD, ptxB, rtxA, tdh*
Stress survival (8)	*clpC, clpP, katA, katB, katG, msrA/B, recN, sodC,*
Exoenzyme (7)	*eta, lip, mpl, nanH, smcL, sspA, stcE*
Invasion (5)	*kpsD, kpsM, kpsT, neuA, neuC*
Biofilm (3)	*algA, algC, luxS*
Antimicrobial activity/Competitive advantage (2)	*acrA, acrB*
Post-translational Modification (1)	*lspA*

### Phylogenetic analysis and SNPs identification

**Figure 4:**
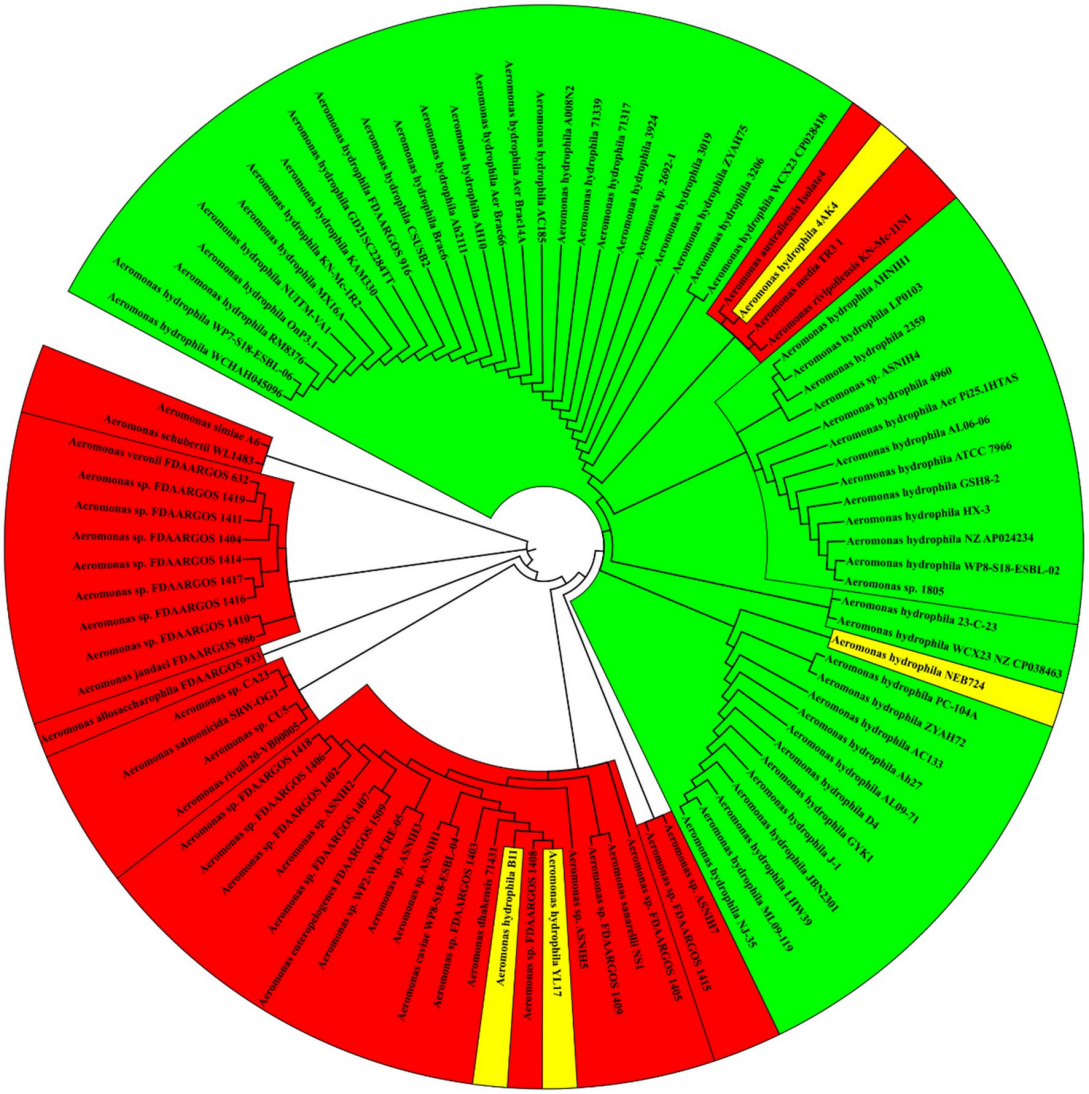
Reconstructed Phylogenetic tree based on 16S rRNA gene (n = 95). The tree was constructed by using the maximum likelihood method and Kimura 2-parameter model^34^ as a best-fit model. All positions with less than 95% site coverage were eliminated. There was a total of 543 positions in the final dataset. Evolutionary analyses were conducted using MEGA-X. Green, valid *A. hydrophila* strains; yellow, strains misidentified as *A. hydrophila*; red, not *A. hydrophila* strains. The tree was visualized using the online iTOL tool^35^.

To investigate the capability of the four remarkable genes to distinguish *A. hydrophila* from other members of the genus *Aeromonas*, phylogenetic analysis was performed and compared to the tree generated using the 16S rRNA sequences. Figure 4 shows that 16S rRNA sequencing is unreliable for discrimination between *A. hydrophila* strains and members of other *Aeromonas* spp*.* It has clubbed mislabeled and non-*A. hydrophila* aeromonads (colored yellow and red respectively) among the valid *A. hydrophila* strains*.* However, when the Roary output was applied to find the differential genes. The phylogenetic tree perfectly aligned with the categorization generated from the ANI analysis based on the whole genome study.

**Figure 5:**
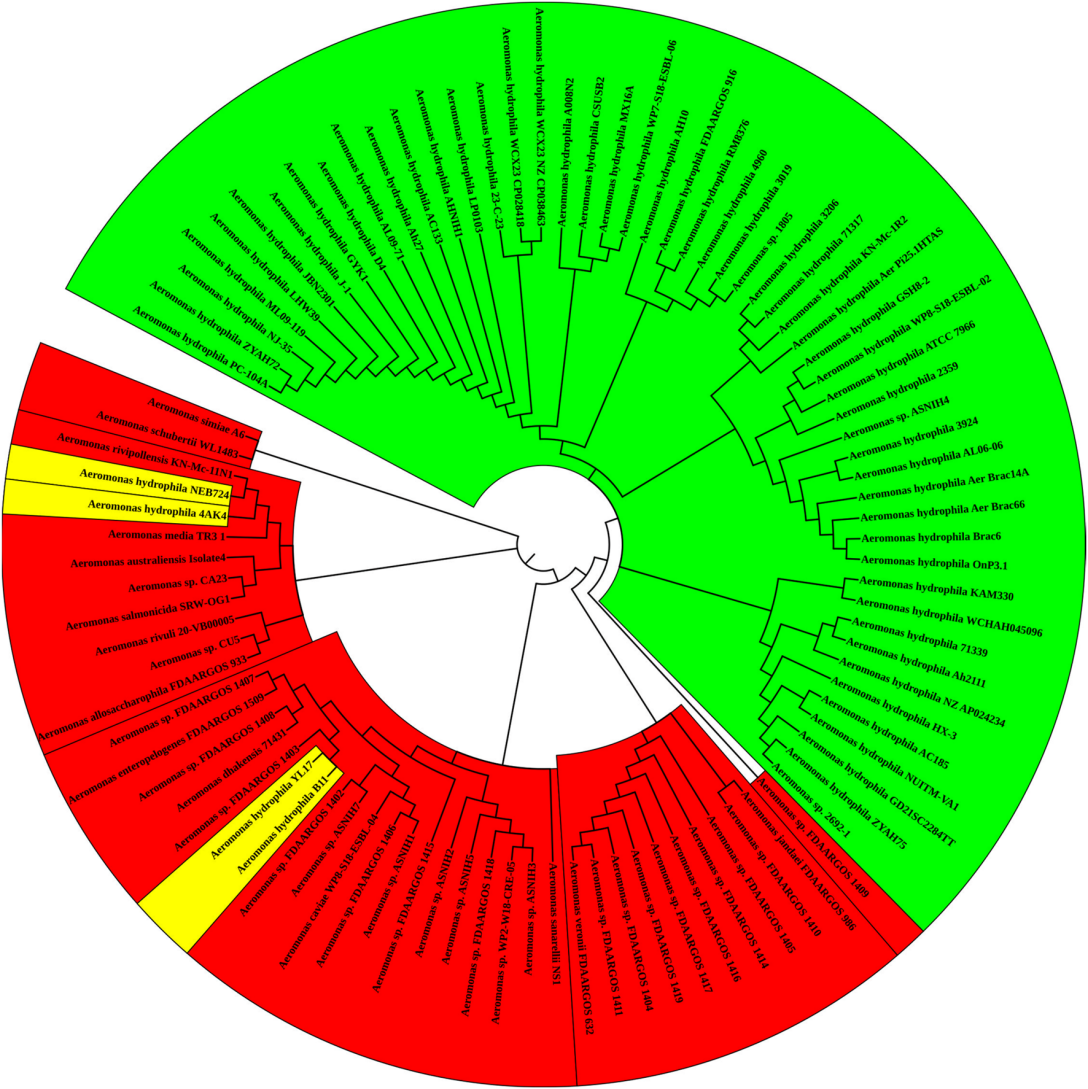
Reconstructed Phylogenetic tree based on the *gmhB* gene (n = 95). The tree was constructed by using the maximum likelihood method and Kimura 2-parameter model^34^ as a best-fit model. All positions with less than 95% site coverage were eliminated. There was a total of 543 positions in the final dataset. Evolutionary analyses were conducted using MEGA-X. Green, valid *A. hydrophila* strains; yellow, strains misidentified as *A. hydrophila*; red, not *A. hydrophila* strains. The tree was visualized using the online iTOL tool^35^.

Accordingly, we propose these four genes as powerful discrimination tools capable of differentiating between *A. hydrophila* and non-*A. hydrophila* aeromonads as ANI value did. Figure 5 shows an example of using one of these genes phylogeny which is perfectly aligned with ANI results.

The name of the proposed genes, as well as the functional annotation of each gene, are listed in Table 2. When categorized phenotypically, they express the functions of immune modulation (*gmhB*), motility (*cheD*), as well as spermidine N(1)-acetyltransferase (*speG*), and maleylpyruvate isomerase (*nagL*). Table 2 also includes a list of the unique SNPs seen in each gene, which could be used to distinguish between *A. hydrophila* and non-*A. hydrophila* aeromonads. Only *speG* was found to have the highest SNP records as it has seven distinct SNPs while each of the other genes has only one unique SNP.

**Table 2:** Differential genes of *A. hydrophila* genomes and their distinctive SNPs.

Genes	Annotation	SNPs unique for *A. hydrophila*
*gmhB*	d-glycero-beta-d-manno-heptose-1,7-bisphosphate 7-phosphatase	88 T>C
*cheD*	Chemoreceptor glutamine deamidase CheD	75 T>G/A/C/-
*speG*	Spermidine N(1)-acetyltransferase	199 G>A, 204 C>T/A/G, 499 A>C/G, 516 G>T/A, 528 T>A/G, 533 G>A, 533 G>A
*nagL*	Maleylpyruvate isomerase	419 C>T/A

## Discussion

The nature of the genus *Aeromonas* which comprises serious pathogens is complex and it is difficult to be identified at the species level through phenotypic characterization^13^. Therefore, it is crucial to explore innovative approaches for the correct taxonomic characterization of its members. The pathogenic species, *A. hydrophila*, is ubiquitous in aquatic environments and is associated with diseases that affect humans and many fish species^36^. The employing of comparative genomics could aid in guidance to detect new biomarkers for identification. Additionally, in silico technologies have made it possible to distinguish between strains thanks to DNA sequences and helped in the development of new molecular markers for the detection and identification of epidemiologically significant microbes^37^. For instance, comparative genomics can reveal insights into evolutionary relationships, gene functions, and molecular mechanisms. However, it has limitations, such as the quality and completeness of genome sequences, which vary between species and databases. Moreover, the annotation and interpretation of genome features vary according to the technology and software used. Furthermore, the evolutionary history and divergence of genomes, and the functional significance and biological relevance of genomic variations might add to its limitations.

The identification of this pathogen can be challenging due to its phenotypic and genotypic diversity and its similarity to other *Aeromonas* species. Misidentification of *A. hydrophila* can lead to incorrect diagnosis, inappropriate treatment, and increased mortality in fish. For human infection, it can be confused with other *Aeromonas* species or other gram-negative bacteria such as *Vibrio*, *Pseudomonas*, or *Escherichia coli*^38^. It can result in increased morbidity and mortality, increased antibiotic resistance, increased healthcare costs, and an increased risk of outbreaks. Therefore, it is important to use reliable and correct methods for the detection and identification of *A. hydrophila*, such as molecular techniques, biochemical tests, and serological assays.

The comparative genomic approach was employed on *Aeromonas* in 2018 by Awan et al.^39^. Even though there were not enough complete genomes available at that time and most were still in the draft stage, they proved that some strains had been incorrectly classified as *Aeromonas hydrophila*. This might be because of using the 16S rRNA as a marker, which can still align the 4AK4 strain to the *A. hydrophila* clade in phylogeny. This incident supplies unequivocal proof that the 16S rRNA sequence cannot be used to name *A. hydrophila* or discriminate it from other species. In addition, the current study revealed that the strains NEB724, B11, and YL17 had previously been mistakenly recognized as *A. hydrophila*. The strain YL17's genome was published in 2016^40^ while, the genomic sequences for NEB724 and B11 were both released in 2020 according to NCBI records. On the other hand, *Aeromonas* sp. 2692-1, 1805, and ASNIH4 were assigned to a particular species, namely, *A. hydrophila*.

Strain misidentification may have a significant impact on the results of diagnostic investigations concerned with microbial pathogens. In a previous study carried out by da Silva Filho et al.^41^ four misidentified *A. hydrophila* strains were eliminated from their analysis. Continuous updating of the NCBI database would reflect these rearrangements to avoid any ambiguity during routine BLAST searches, as well as primer or probe design for diagnostics or epidemiological applications.

Pathogenomics is a field of science that uses genomics research to assess the pathogenicity potential of bacteria. However, most of the published epidemiological studies consider only a few genes^13,18,42^. In the current study, 312 virulence genes were detected in the *A. hydrophila* pan-genome. The existence of these genes in *A. hydrophile’*s pan-genomes adds to the complexity of this significant disease-causing organism and makes it crucial to accurately diagnose the etiological agent. This all, in light of most of the virulence genes identified in the literature, was carried out on a former *A. hydrophila* SSU strain, which has been reclassified later as *Aeromonas* dhakensis^19^. The present investigation explored four genes (*gmhB*, *cheD*, *speG*, and *nagL*) in all strains of *A. hydrophila*. These genes show great similarity among the recognized *A. hydrophila* strains and significant dissimilarity among other genomes. Consequently, these uncovered genes may provide a potential genotyping tool for distinguishing *A. hydrophila* strains from other aeromonads.

The phylogenetic analysis of these genes has shown the same trend as ANI, suggesting a straightforward strategy for *A. hydrophila* based on single gene sequences rather than the MLST approach, or MLST combined with other methods as recommended by Beaz-Hidalgo et. al.^14^. Using a single, properly chosen gene for microbial typing has the advantages of being fast, cheap, and efficient. Furthermore, SNPs were proposed for accurate identification of *Bacillus* spp. responsible for food poisoning^43^. Similarly, the distinct SNPs discovered in the current study are suggested as a good starting point to develop a novel molecular typing method that precisely names *A. hydrophila* strains. Similar methods have been successfully applied for *Salmonella* investigations since some genes have been discovered as markers present in certain serovars^44,45^.

## Conclusion

This work presents a recent comparative study of the accessible genomes of *A. hydrophila*. Using the most recent genomes and virulence factor databases. The study was expanded to find the pan-virulence genes and assess the pathogenic potential of *A. hydrophila*. Four distinct genes that could be used as molecular markers for *A. hydrophila* identification were discovered. These findings broaden our knowledge and serve as a starting point for an in-depth investigation of the pathogenicity and virulence of *A. hydrophila*. Most of the discovered genes engage in immune system modulation, microbe motility, or microbial secretion systems, which raises questions about their role in the pathogenesis of *A. hydrophila* in fish and human hosts.

## Supplementary Information

Supplementary Table S1.

Supplementary Table S2.

Supplementary Table S3.

## Data Availability

The datasets used in the investigation are accessible from the NCBI, and complete information, including the accession number and download links, is included in the supplementary Table S1. Any additional information requested is available upon request from the corresponding author.
